# Beyond the Meal: Trophic Controls by Pelagic and Demersal Chondrichthyes in Two Different Mediterranean Marine Food Webs

**DOI:** 10.1002/ece3.72540

**Published:** 2025-11-19

**Authors:** P. Ricci, L. Finotto, A. Barausse, C. Zampieri, C. Mazzoldi, G. Cipriano, F. P. De Luca, R. Carlucci

**Affiliations:** ^1^ Department of Biology University of Padua Padua Italy; ^2^ National Interuniversity Consortium for Marine Sciences, CoNISMa Rome Italy; ^3^ National Biodiversity Future Center, NBFC Palermo Italy; ^4^ Department of Biosciences, Biotechnology and Environment University of Bari Bari Italy

**Keywords:** apex predators, cascading effects, Ecopath, Northern Ionian Sea, top‐down controls, trophic levels

## Abstract

Pelagic and demersal Chondrichthyes can assume different patterns of trophic controls on marine food webs, sustaining the functioning of marine ecosystems. These species are impacted by fisheries requiring conservation measures to mitigate the loss of their ecological roles. Amass‐balanced modelling approach based on the Ecopath routine was adopted to investigate the trophic roles exhibited by Chondrichthyes through a comparative analysis of two food webs (Calabrian and Salento) within the Northern Ionian Sea (Central Mediterranean Sea). A total of 10 functional groups (FGs) of pelagic (3) and demersal (7) Chondrichthyes were represented in the models. Five ecological indicators were adopted in the analysis of Chondrichthyes: fractional trophic levels (TL) and their variance expressed by the Omnivory index; the importance of FGs as keystone species through the keystoneness indices and their trophic controls played on discrete TLs through the Mixed Trophic Impact analysis; the exploitation rates of 7 fishing gears and their direct and indirect impacts on target groups. Pelagic sharks showed high TLs and a generalist trophic spectrum, except for the planktivorous basking shark (TL = 3.2). Changes in their importance as keystone species between demersal and pelagic sharks were observed between the two investigated areas affected by different ecosystem traits. The blue shark exerted direct top‐down controls on their prey located in the fourth TL, while the kitefin shark (demersal apex predator) showed positive top‐down cascading impacts with indirect effects on the FGs of the lower trophic levels. Demersal elasmobranchs played the role of meso‐predators, exhibiting negative and positive effects in both food webs. Bottom trawl and drifting longline showed the most negative direct and indirect impacts on demersal and pelagic elasmobranchs, respectively, and fishing overexploitation was estimated for pelagic and bathyal demersal sharks Conservation measures are required to protect these species and their ecological roles in marine ecosystems.

## Graphical abstract

Pelagic and demersal sharks played different top‐down controls in the food web. Pelagic sharks act as apex predators impacting mesoconsumers. Trophic controls patterns by demersal sharks change according to ecosystem traits.

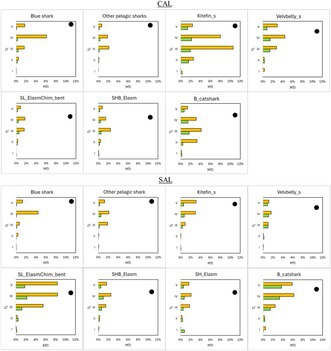

## 1 Introduction

The public perception of sharks is that they are often associated with ferocious pelagic predators, voracious and capable of consuming even large prey. However, human understanding of the ecological role of sharks and other chondrichthyans has changed throughout history, nowadays shifting toward an awareness of their multiple and complex ecological roles in marine ecosystems (Mazzoldi et al. 2019; Giovos et al. 2021; Dedman et al. 2024). Indeed, beyond the food consumed by sharks, there is a complex dimension of direct and indirect interactions, including both trophic (direct predation) and non‐trophic (such as risk effects on prey behaviours) processes (see Dedman et al. 2024). These ecosystem processes exhibited in pelagic and demersal domains can contribute to the stability of marine ecosystems and their biodiversity, as well as the provision of ecosystem services enjoyed by humans (Hammerschlag et al. 2019). For instance, large sharks at the apex of the food web can have effects of controls on herbivores contributing to macroalgal establishment and seagrasses biodiversity in the coastal areas of Western Australia (Nowicki et al. 2021). The loss of apex sharks can alter the stability of trophic cascades with excessive increases of mesoconsumers in temperate marine ecosystems (Ferretti et al. 2010), as well as in coral reefs, when specific conditions of multiple interactions with other predators occur (Roff et al. 2016). In this context, the ecological role of sharks is also linked to carbon sequestration by primary benthic producers, supporting part of climate regulation, although quantitative studies on these aspects are still scarce (Dixon and Gallagher 2023). Thus, a full quantification of the ecological roles of these species is essential to understand the consequences of their conservation and restoration for marine biodiversity, ecosystems, and fisheries. Indeed, elasmobranchs are highly vulnerable to fishing impacts that represent one of the most important threats for these animals (Giovos et al. 2024).

Studying the trophic role played by Chondrichthyes is a challenging task in large marine systems, given the high mobility of these elusive organisms and the complexity of these ecosystems. Therefore, investigations into the trophic niche of single species have recently been conducted through the combination of traditional and emerging techniques, such as stomach content, stable isotope and DNA metabarcoding analyses (Clark et al. 2023; Cicala et al. 2024), while the quantification of trophic controls and interactions with fisheries requires the adoption of trophodynamic modelling approaches. In particular, the trophic dynamics of these predators have often been investigated through mass‐balanced food‐web models (Ecopath with Ecosim, Christensen and Walters 2004), aiming to define their ecological roles and trophic controls, as well as their interaction with fishing gears (Kitchell et al. 2002; Bornatowski et al. 2017; Rupp and Bornatowski 2021; Corrales et al. 2022). Different patterns occur in top‐down controls played by pelagic and demersal sharks in oceanic ecosystems, but the clear identification of these controls is a challenge (Baum and Worm 2009; Dedman et al. 2024; Storm et al. 2025). Indeed, top‐down controls exhibited by elasmobranchs in complex ecosystems are ranged among several kinds of interaction mechanisms, such as direct predation, risk effects and indirect mechanisms, such as trophic cascades. In this framework, sharks and rays tend to assume roles of macropredators or meso‐predators, but changes in their roles are also affected by spatial and temporal factors (Ferretti et al. 2010, 2013). Therefore, it is not always easy to identify the ecological roles played and the strength of the trophic controls exerted by these predators.

In the Mediterranean region, elasmobranchs are considered important keystone predators mainly threatened by fishing impacts (Piroddi et al. 2015). In particular, the percentage of species threatened with extinction has risen to 64.4% (Dulvy et al. 2016). Moreover, for cosmopolitan species, the Mediterranean populations are usually characterised by a worse conservation status compared to their respective oceanic counterparts. This is the case of four pelagic species studied here: the blue ( *Prionace glauca* ), thresher ( *Alopias vulpinus* ), and shortfin mako sharks ( *Isurus oxyrinchus* ) are respectively assessed as being near threatened, vulnerable, and endangered globally, while they are considered critically endangered, endangered, and critically endangered in the Mediterranean Sea (Dulvy et al. 2021). The conservation status of the basking shark ( *Cetorhinus maximus* ) is critical, and the species is assessed as being endangered both globally and in the Mediterranean area.

Overall, the trophic roles and interactions of pelagic sharks have been scarcely investigated in comparison to demersal species in the Mediterranean Sea (Coll and Libralato 2012; Seyer et al. 2023), while detailed analyses are available for the bathyal demersal elasmobranchs in the Calabrian subregion in the Northern Ionian Sea (NIS, Central Mediterranean Sea) (Ricci et al. 2021). Importantly, food‐web models analysed in very proximal areas within the NIS, such as Salento, stressed differences in trophic structure, ecological roles of top predators, and fishing effort patterns (Ricci et al. 2019, 2023). However, comparisons of the differences in the ecological role between pelagic and demersal sharks have not yet been explored in depth. Therefore, this study develops a comparative analysis between pelagic and demersal Chondrichthyes, adopting as a case study two different food webs modelled in NIS through the well‐known Ecopath approach in the period 2013–2015. Several ecological and fishing indicators were used to define trophic levels, patterns of trophic controls in ecological domains, and interactions with fishing gears.

## 2 Materials and Methods

### 2.1 Study Area

**FIGURE 1:**
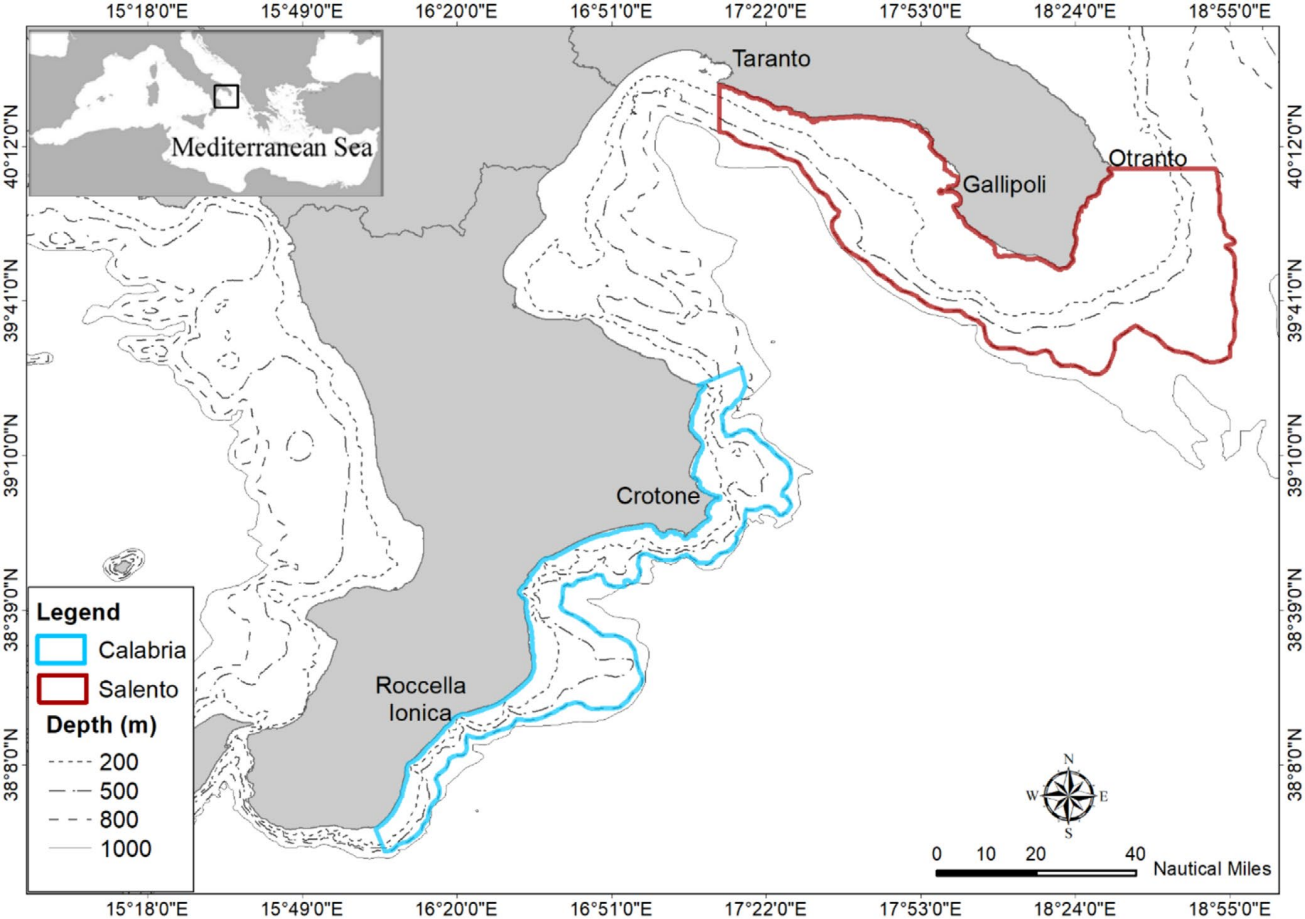
Map of modelled areas in the Northern Ionian Sea with Salento (blue) and Calabria (red).

The Northern Ionian Sea (NIS) has complex geomorphology and oceanography traits and oligotrophic waters, with differences along the latitudinal gradient moving from the north‐eastern zone (Salento, SAL) to the south‐western one (Calabria, CAL) (Ricci et al. 2019, 2022, and references therein). The former area is characterised by a broad continental shelf, while the latter shows a high density of submarine canyons close to the coastline. Moreover, temporal changes in the deep‐water circulation and oceanographic traits are widely documented in the NIS basin (Lavigne et al. 2018), as well as their multiple effects on abundances and the spatial distribution of demersal and benthopelagic species (Civitarese et al. 2010; Carlucci et al. 2018). The spatial distribution of demersal Chondrichthyes and temporal changes in abundance and biomass are well studied in the NIS region, highlighting different distributions and abundances of species between the SAL and CAL areas (Sion et al. 2024 and reference therein). Thus, the areas modelled in this study are included in a range of depth between 10 and 800 m, covering surfaces of 6660 and 3649 km^2^ in the SAL and CAL zones, respectively (Figure 1).

### 2.2 Modelling Approach

The structure and functioning of the food webs were described through a mass‐balance model approach based on open‐source software Ecopath with Ecosim v.6.6.5 (www.ecopath.org, Christensen et al. 2008). Ecopath provides a static snapshot of the food web described by functional groups (FGs, i.e., nodes of the web composed of one or more species with a trophic similarity), which follow the assumption of an energy balance within a quasi‐steady state. Links between FGs are formally described by a system of linear equations that describe the trophic fluxes for each FG through production and consumption terms (Christensen et al. 2008). In particular, the variables for each FG_*i*_ are the Biomass B_i_, production rate (P/B_*i*_), consumption rate (Q/B_*i*_), diet composition (DC_*ij*_), unassimilated food (U/Q_*i*_), catches (Y_*i*_) and exports (*E*_*i*_). The solution of the linear system is solved by Ecopath which allows the estimation of one unknown parameter per equation and FG, either B, P/B, Q/B, or EE. Further details are reported in the literature (Christensen et al. 2008; Heymans et al. 2016).

### 2.3 Functional Groups and Fishing Gears

Considering the availability of an original and detailed model for the Calabrian area in the 2013–2015 years (Ricci et al. 2021), this period was selected for the implementation of both updated SAL and CAL models. The CAL model was used as a starting model to develop a similar model in the SAL area, modifying the original model structure to better describe pelagic sharks and some prey groups. The descriptions of some new pelagic shark FGs have been added to the original model and the representation of some FGs has been improved. The setting of this new model of the Calabrian area was applied in the same manner to the Salento area, using a total of 60 FGs (Table 1; Table S1). The three new FGs groups of pelagic sharks are: *P. glauca* (blue shark, FG 6), *C. maximus* (basking shark, FG 7), *A. vulpinus* and *I. oxyrinchus* (Other pelagic sharks, FG 8). In addition, *taxa* belonging to the Mackerels (*Trachurus* spp., and *Scomber* spp.) have been separated from the previous group of Medium pelagic fishes, becoming a single FG (28) to reduce the cannibalism of the group, according to the smaller size of mackerels and their occurrence as prey in the diets of the most abundant species grouped in FG 29, such as *Seriola dumerili* (Andaloro and Pipitone 1997), *Coryphaena hippurus* (Massutí et al. 1998), *Sarda sarda* (Genç et al. 2019), *Pomatomus saltatrix* (Mancini et al. 2022). Moreover, the original FGs Slope Fishes planktivorous and Slope Fishes crustaceans‐feeders have been aggregated into a unique FG, given their trophic similarity. Notably, the species aggregation into FGs is based on the similarity in feeding habits, bathymetric positions and faunal group traits (Ricci et al. 2021). Thus, the codification of FG names reports the bathymetric position, such as shelf (SH), Shelf‐Break (SHB), and Slope (SL) for groups belonging to benthic, demersal or benthopelagic domains, and the main feeding habits of the group (e.g., piscivorous, generalist, planktivorous, etc.) in the last part.

**TABLE 1:** Functional groups and code (FG name) and their ecological domains (Dom.) used in the two food‐web models.

no.	FG name	Code	Dom.	no.	FG name	Code	Dom.	no.	FG name	Code
1	Odontocetes	Odontocetes	PEL	30	Macrourids	Macrourids	DEM	59	Discards	Disc
2	Fin whales	F whale	PEL	31	Myctophids	Myctophids	BP	60	Detritus	Det
3	Loggerhead turtles	Log turtle	PEL	32	Red mullet	R mullet	DEM			
4	Seabirds	Seabirds	PEL	33	Hake	Hake	DEM			
5	Large pelagic fishes	L pelagics	PEL	34	Anglers	Anglers	DEM			
**6**	**Blue shark**	**Blue shark**	**PEL**	35	Slope Squids	SL_Squids	BP			
**7**	**Basking shark**	**Basking shark**	**PEL**	36	Shelf Squids	SH_Squids	BP			
**8**	**Other pelagic sharks**	**Other pelagic sharks**	**PEL**	37	Shelf Cephalopods	SH_Cephalopods	BENT			
**9**	**Slope Elasmobranchs Chimeras benthic feeders**	**SL_ElasmChim_bent**	**DEM**	38	Slope Cephalopods	SL_Cephalopods	BENT			
**10**	**Shelf‐Break Elasmobranchs**	**SHB_Elasm**	**DEM**	39	Shelf‐Break Bobtail Squids	SHB_Bob Squids	BP			
**11**	**Shelf Elasmobranchs**	**SH_Elasm**	**DEM**	40	Benthopelagic Shrimps	Shrimps_BP	BP			
**12**	**Slope Elasmobranchs fish‐feeders**	**SL_Elasm_pisc**	**DEM**	41	Slope Decapods scavengers	SL_Decapods_scav	BENT			
**13**	**Kitefin shark**	**Kitefin_s**	**DEM**	42	Slope Crabs	SL_Crabs	BENT			
**14**	**Velvet belly lanternshark**	**Velvbelly_s**	**DEM**	43	Shelf Crabs	SH_Crabs	BENT			
**15**	**Blackmouth catshark**	**B_catshark**	**DEM**	44	Deep‐water Rose Shrimp	DWR Shrimp	DEM			
16	Demersal opportunistic fishes	DEM opportunistic fishes	DEM	45	Red Giant shrimp	RG Shrimp	DEM			
17	Slope Demersal fishes generalist‐feeders	SL_DEM fishes_gen	DEM	46	Red and Blue shrimp	RB Shrimp	DEM			
18	Shelf Demersal fishes generalist‐feeders	SH_DEM fishes_gen	DEM	47	Polychaetes	Polychaets	BENT			
19	Shelf Demersal fishes fish‐feeders	SH_DEM fish_pisc	DEM	48	Macrobenthic invertebrates	Macrobent inv	BENT			
20	Slope Bathypelagic fishes fish‐feeders	SL_BP fishes_pisc	BP	49	Gelatinous plankton	Gel plank	PEL			
21	Slope Demersal fishes Decapods‐feeders	SL_DEM fishes_Bent crust	DEM	50	Suprabenthic crustaceans	Supbent crust	BENT			
22	Slope Fishes crustaceans‐feeders	SL_BP fishes_crust	BP	51	Macrozooplankton	Macrozooplank	PEL			
23	Shelf‐Break Demersal fishes crustaceans‐feeders	SHB_DEM fishes_Bent crust	DEM	52	Mesozooplankton	Mesozooplank	PEL			
24	Shelf Demersal fishes benthic crustaceans‐feeders	SH_DEM fishes_Bent crust	DEM	53	Microzooplankton	Microzooplank	PEL			
25	Shelf Demersal fishes benthic invertebrates‐feeders	SH_DEM fishes_Bent inv	DEM	54	Bacterioplankton	Bactplank	PEL			
26	Shelf‐Break Demersal fishes planktivorous	SHB_DEM fishes_plankt	DEM	55	Seagrasses and algae	Seagrasses‐algae	BENT			
27	Small pelagic fishes	S pelagics	PEL	56	Large phytoplankton	L phytoplankton	PEL			
28	Mackerels	Mackarels	PEL	57	Small phytoplankton	S phytoplankton	PEL			
29	Medium pelagic fishes	M pelagics	PEL	58	Suspended Particulate Organic Matter	SPOM				

*Note:* Dom. assigned were Pelagic (PEL), Benthopelagic (BP), Demersal (DEM) and Benthic (BENT). Chondrichthyes FGs are in bold.

The fishing gears represented in both models are the otter bottom trawl (OTB), drifting longline (LLD), setting longline (LLS), passive nets (GNX), mixed gears (MIX) and purse seine (PS).

### 2.4 Chondrichthyes FGs: Input Data and Parametrization

Inputs for demersal Chondrichthyes in the CAL model were acquired from another model (Ricci et al. 2021), while the SAL model was realized by adopting the same data sources and procedures used for the building of the original CAL model.

In the SAL model, biomass data of demersal Chondrichthyes (expressed as biomass indices, kg km^−2^) were obtained from 21 annual experimental trawl hauls, conducted within the ‘MEDiterranean International Trawl Survey’ (MEDITS) research program (Spedicato et al. 2019). These relative biomass data were transformed into t km^−2^, corrected through a catchability factor by species, adopting the approach used in the CAL model, and successively aggregated into corresponding FGs as the sum of each (Ricci et al. 2021). All demersal Chondrichthyes FGs were characterised by a biomass value, except for the SL_Elasm_pisc FG, for which the final biomass was estimated by fixing the EE according to the value obtained from the CAL balanced model (0.769; Christensen et al. 2008). Production (P/B) and Consumption (Q/B) rates were obtained from previous estimates reported in local models from the NIS (Carlucci et al. 2021; Ricci et al. 2022). P/B and Q/B rates were aggregated into FGs by averaging biomass‐weighted values using biomasses from MEDITS (period 2013–2015). The DC matrix used for each model was obtained by Ricci et al. (2021), except for *Dalatias licha* , which was updated through the stomach contents data from the Strait of Sicily (Calabrò et al. 2024).

For pelagic sharks, inputs were obtained from several data sources (other models, observation data, open databases) or calculated from empirical equations (for more details see Appendix S1). In the SAL model, data of biomass for the basking shark were obtained from the total lengths associated with abundance data acquired by opportunistic sightings in the period 2011–2014 (Carlucci et al. 2014; De Sabata et al. 2013). From total lengths, wet weights of 14 individuals (5 in 2011, and 9 in 2013; Appendix S1) were calculated adopting the length‐weight relationship for the Mediterranean basking sharks (Mancusi et al. 2020, see Appendix S1), and final input biomass in the model was obtained by summing up the weight within the single year, averaging between the 2 years considered, and standardising for the surface of the SAL model. Sightings were not available for the basking sharks in the CAL area; therefore the biomass was estimated by fixing the EE value (0.61) according to that obtained from the final balanced SAL model (Table 2).

**TABLE 2:** Parameters of pelagic sharks and references used to estimate final input data in the NIS Ecopath models: growth rate (K), asymptotic length (L_inf_), natural mortality (M, from the equation of Pauly 1980), fishing mortality rate (F), total mortality rate (Z), Ecotrophic Efficiency (EE), Consumption rate (Q/B, from Palomares and Pauly 1999).

Species (FG)	*K*	L_inf_ (cm)	Ref.	*M*	*F*	Ref.	Z = P/B	EE	Q/B	Ref.
Blue shark (6)—CR	0.13	401.5	Megalofonou et al. 2009	0.192	0.22	Musyl and Gilman 2019	0.409	0.530	3.288	Kitchell et al. 2002
Basking shark (7)—EN	0.062	1000	Pauly 2002	0.081	—	Pauly 2002	0.193	0.61^a^	9.039	Sims 2008 (T°C corrected for NIS)
Thresher shark (8)—VU	0.13	451.7	Gervelis and Natanson 2013	0.186	0.34	Musyl and Gilman 2019	0.526	0.647	2.44	Palomares and Pauly 1999
Mako shark (8)—CR	0.06	368.2	Barreto et al. 2016	0.119	0.22	Musyl and Gilman 2019	0.339	0.65	3.934	Bornatowski et al. 2018
Other pelagic sharks (FG8, mean values)	—	—	—	0.152	0.280	—	0.432	0.648	3.187	—

*Note:* More details are reported in Appendix S1. IUCN Codes on the conservation status (CR, Critically Endangered; EN, Endangered; VU, Vulnerable) at the Mediterranean level are reported.

^a^ EE value adopted for FG6 in the CAL model, while a biomass value (0.001 t km^−2^ year^−1^) was used in the SAL one.

For the groups of the blue shark and other pelagic sharks, biomass or other abundance data were not available; thus EE values were fixed to estimate the final input biomass (Table 2). These values were estimated assuming that these three sharks are not affected by predation mortality, being apex predators in the food web, and only experience natural (*M*) and fishing mortality (*F*). Thus, these mortality components can be used to represent EE, which is the proportion of the production of each species consumed in the system (Christensen and Walters 2004). *F* values were obtained from estimates provided by a random‐effect meta‐analysis on post‐release fishing mortality data at a global level (Musyl and Gilman 2019) and reported in Table 2. This data source was chosen to provide input data with lower uncertainty to the models (see Appendix S1 for more details). Indeed, estimates of F values are not available for these pelagic predators in the modeled areas, and the few data available in the Ionian region are very dated and based on proxy values, such as *F* values represented as Catch Per Unit Effort of several fishing gears calculated for blue, mako and thresher sharks in the Eastern Ionian Sea (1998–2001; Megalofonou et al. 2005).

In the ecological conditions characterised by a dynamic equilibrium, the P/B rate is equal to the total mortality rate (*Z*), estimated by the sum of *M* and *F* mortality components (Allen 1971). Therefore, *M* values of pelagic sharks were calculated by means of the empirical equation of Pauly (1980), based on the annual growth rate (*K*) and the asymptotic length (L_inf_, in cm) estimated from the Von Bertalanffy growth equation, and the mean temperature of the water (in °C) recorded for the period in which the species occurs in the study area (for more details on the calculation see Appendix S1). Then, *F* and *M* values were summed to obtain final P/B rates of each target FG (Table 2).

The Q/B rates for the pelagic sharks were obtained using empirical relationships (Palomares and Pauly 1999; Sims 2008) or models (Bornatowski et al. 2018; Kitchell et al. 2002), based on growth parameters and water temperatures (Appendix S1).

The main parameters used to calculate the input data (P/B, Q/B, EE) for the pelagic sharks are reported in Table 2.

Quantitative information and sources on diets of pelagic sharks are reported in Appendix S1.

### 2.5 Other FGs and Fishing Gears: Input Data, Parametrization and Balancing

Data for all remaining demersal and benthopelagic FGs for the CAL model were acquired from the original model (Ricci et al. 2021), and the data inputs for these FGs in the SAL model were elaborated according to the procedures used for the Chondrichthyes FGs (see Appendix S1).

Recently updated official fishery data (landings and bycatch, in annual kg) acquired from the Italian National Fisheries and Aquaculture Economic Research Institute (NISEA), were used to represent the local fishing gears (see previous paragraph 2.3). Data were obtained as Ionian Apulian and Calabria subregions for the period 2005–2021. In the case of missing data for a given species during the modelled period (2013–2015), the average value of the time series was applied. Data were successively standardised for the surface of each modelled area in t km^−2^ and summed in the corresponding FG. Discards derived from commercial *taxa* were calculated using discard rates by gears and *taxa* acquired from several data sources (Tsagarakis et al. 2014; Sartor et al. 2016). The discard fraction for non‐commercial *taxa* caught by OTB was estimated based on the proportion of commercial and non‐commercial discards in MEDITS data for the period of investigation (see Ricci et al. 2019).

All species aggregated into FGs, inputs (B, P/B, Q/B, EE), diets, and landings and discard data used in both models are reported in (Tables S1–S4).

The balancing of both models was conducted by applying the standard procedure based on the check and adjustment of input data coherently with basic thermodynamic laws, and ecological rules and principles at the ecosystem level (Heymans et al. 2016). The balancing procedure is detailed in Appendix S1.

### 2.6 Ecological and Fishing Indicators Analysis

A comparative analysis to explore the ecological role of Chondrichthyes in food‐web models was performed using ecological and fishing indicators: Trophic Level (TL), Omnivore index (OI), relative Overall Effect (relOE) and Keystoneness indices (KS, Libralato et al. 2006; Valls et al. 2015), and the Mixed Trophic Impact analysis (MTI, Ulanowicz and Puccia 1990). The description of TL and OI is reported widely in the literature (see Ricci et al. 2021 and references therein). A comparative analysis on TL, OI and relOE was carried out by plotting the estimated indicator values for each FG of both models in a graph, with the SAL model values (*x*‐axis) and CAL model values (*y*‐axis), respectively. Thus, FGs with the same values of ecological indicators in both models would fall on the diagonal lines of plots, while FGs distant from the diagonal would indicate differences between the two models. Differences between the values of ecological indicators estimated by models were considered relevant when a FG_*i*_, represented by a point *P*(*x*_*i*_, *y*_*i*_) (where *x*_*i*_ and *y*_*i*_ represent the value of the given indicator in the two models), showed a perpendicular distance (D) from the diagonal line higher than the 75th percentile of the *D*‐values distribution calculated for that ecological indicator. D between the diagonal line and *P*(*x*_*i*_, *y*_*i*_) was calculated as follows:

D=xi−yi√2

where *x*_i_ and *y*_*i*_ are SAL and CAL values of a given ecological indicator for FG_*i*_.

The MTI analysis is based on the quantification of the relative direct and indirect impact due to the biomass change of a group (impacting FG_*i*_) on each of the other groups (impacted FG_*j*_) in the food web, including fishing gears (Table S5). Thus, positive/negative impact (*m*_*ij*_ with *i* = impacting FG, and *j* = all other impacted FGs) corresponds to an increase/decrease in biomass of the FG*j* due to a slight increase in biomass of the impacting FG_*i*_. Therefore, negative impacts can be associated with prevailing top‐down controls and positive ones with bottom‐up controls (Libralato et al. 2006). The different *m*_*ij*_ terms in the MTI matrix for a FG_*i*_ can be used to compute the Overall Effect (OE_*i*_) exerted on all the other FGs_*j*_:

$${\mathrm{OE}}_{i}=\sqrt{\sum_{j=1}^{n}m_{\mathrm{ij}}^{2}}$$

where the impact on the group itself (*m*_*ij*_ with *i* = *j*) is not considered (Libralato et al. 2006).

The relative OE_*i*_ (calculated as a relative value with respect to the maximum OE value) and relative biomass (p_i_) of a FG_*i*_ in the food web (excluding detritus groups) allow the calculation of the importance of each FG as a keystone species, adopting two indices in the analysis: KS1 (Libralato et al. 2006) and KS3 (Valls et al. 2015).

Trophic controls were analyzed considering the direct and indirect impacts (or effects, DirOE and IndOE) by Chondrichthyes, OEs exerted on FGs aggregated in ecological domains, the top‐down effect (td%) by Chondrichthyes calculated as the proportion of negative MTI values to the total MTI ones (expressed in % value; Coll and Libralato 2012), and the comparison of negative and positive OEs exerted along discrete TLs of food webs. Such analysis is based on the aggregation of MTI values of impacted FGs within several aggregation levels.

DirOE and IndOE for each Chondrichthyan FG were calculated to quantify the importance of predation in the trophic regulation, as the percentage contribution in each food web. DirOE corresponded to MTI values of prey reported in the diet of each Chondrichthyes FG.

Trophic impacts on ecological domains were calculated as percentage values of Chondrichthyes in each food web, aggregating impacted FGs into the following domains: Benthic (BENT), Demersal (DEM), Benthopelagic (BP), and Pelagic (PEL) (Table 1).

The comparison of top‐down controls along TLs adopted the approach of Carlucci et al. (2021). The positive (Pos) and negative (Neg) MTI values of each Chondrichthyes FG were separately used to estimate impacts exerted on discrete TLs by weighting each FG's impact (*m*_*ij*_ with *i* = Chondrichthyes and *j* all other FGs) through the proportion of flows of group *j* belonging to integer TLs. Proportions of the flows by discrete TLs were obtained using the Trophic Level Decomposition routine estimated by the Ecological Network Analysis routine (Christensen et al. 2008). The sum weighted MTI for each group *i* was calculated for both Pos and Neg values in each discrete TL as percentage values (%) of the total MTI. Chondrichthyan FGs with the highest MTI% are reported.

Fishery impacts on Chondrichthyan FGs were assessed through the exploitation rate (*F*/*Z*) and MTI of fishing gears. The *F*/*Z* rate indicates the proportion of fishing mortality (*F*) to the total mortality (*Z*) for each of the FGs. The *F*/*Z* values were compared to the threshold limit of 0.50, where values higher than this limit highlight a potential overfishing condition for a given resource (Kolding et al. 2015).

The ecosystems' traits of the two food webs have been described using a total of 19 ecological indicators based on thermodynamical principles and the theory of ecosystem development formulated by Odum (1969) and integrated into the EwE framework by Christensen (1995). Definitions and descriptions of indicators are reported in Appendix S1.

## 3 Results

**FIGURE 2:**
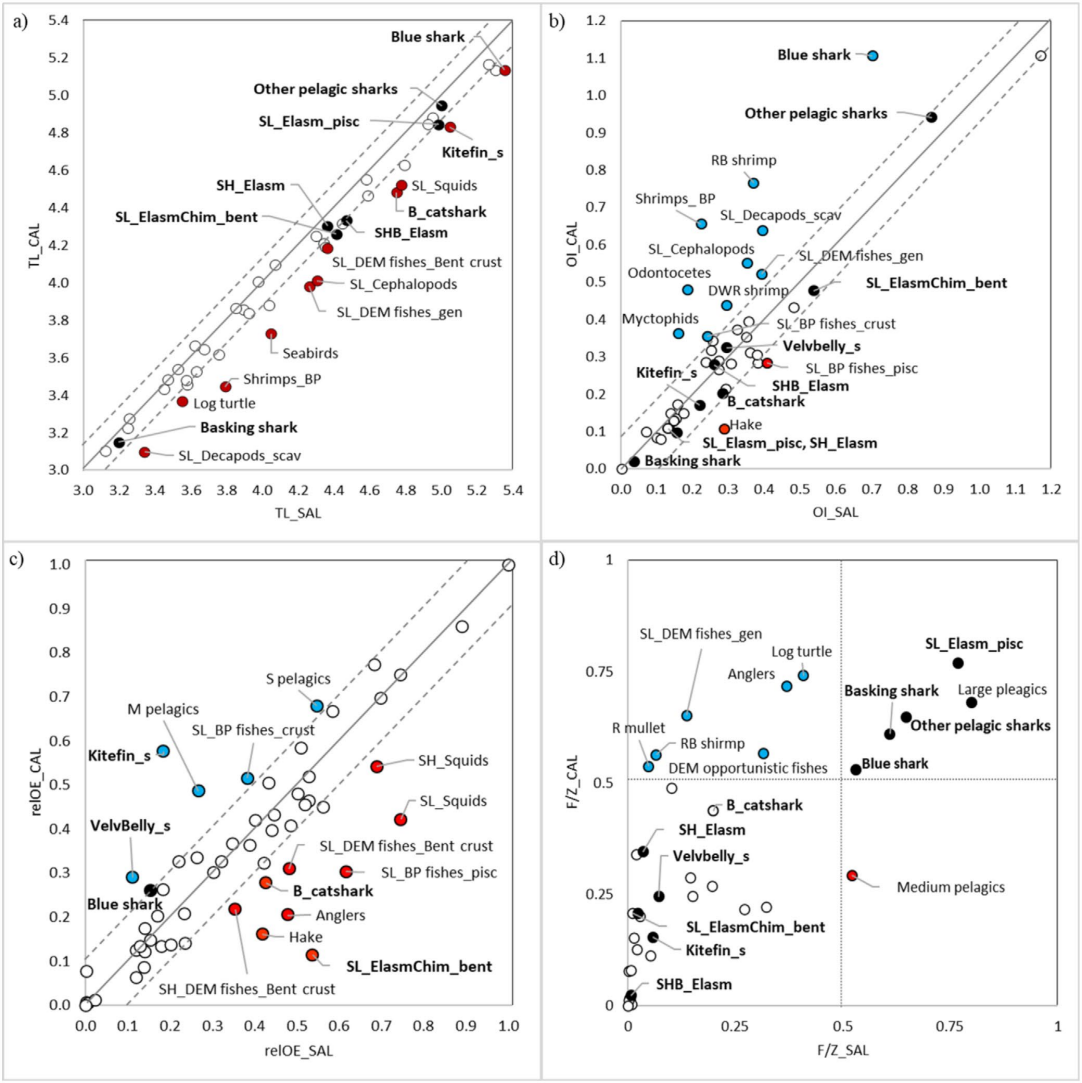
Estimations of (a) Trophic levels (TL), (b) Omnivory index (OI), Overall Effect (OE), and (d) Exploitation rate (E) for the Salento (SAL) and Calabria (CAL) FGs. Circles showing relatively equal values in both food webs are black‐coloured for Chondrichthyes FGs and white‐coloured for others. FGs with a higher value, that is, over the dashed lines around the diagonal line, for a food‐web indicator in one of the two webs are either red (Salento) or blue‐coloured (Calabria). In each panel (a–c), the distance between either dashed line and the diagonal is equal to the 75th percentile of the distribution of the distances between each FG and the corresponding diagonal (solid line). In panel (d), dotted lines mark the *F*/*Z* rate = 0.50.

Trophic levels estimated by the model for all FGs indicated the occurrence of values higher in the SAL food web than that in the CAL one (Figure 2a). The main difference was observed for the FGs of medium‐high TLs, and the blue and kitefin sharks, the B_catshark and the Velvbelly_s, which showed the highest variations of TL values between the CAL and SAL models. Other pelagic sharks and the Basking shark showed similar TL values in both food‐web models.

The blue shark showed a higher OI value in the CAL food web than the SAL one (Figure 2b). In addition, high OI values were estimated for the other pelagic sharks in both areas. The basking shark showed the highest level of feeding specialisation (OI < 0.05), followed by SL_Elasm_pisc, SH_Elasm, Kitefin_s, SHB_Elasm, and B_catshark (0.10 < OI < 0.30), but no relevant differences in the OI values between the two models were estimated.

Considering the trophic impacts of Chondrichthyes (expressed as relative Overall Effect, relOE), the Kitefin_s and Velvbelly_s played high impacts in the CAL food web, while SL_ElasmChim_bent and B_catshark in the SAL one (Figure 2c). Overall, demersal fish groups (hake, frogfish, squid and demersal fish feeding on crustaceans) had a greater trophic impact in the SAL food web than in the CAL food web, where pelagic fish groups had a greater impact.

### 3.1 Ecological Role and Trophic Impacts of Chondrichthyes

**FIGURE 3:**
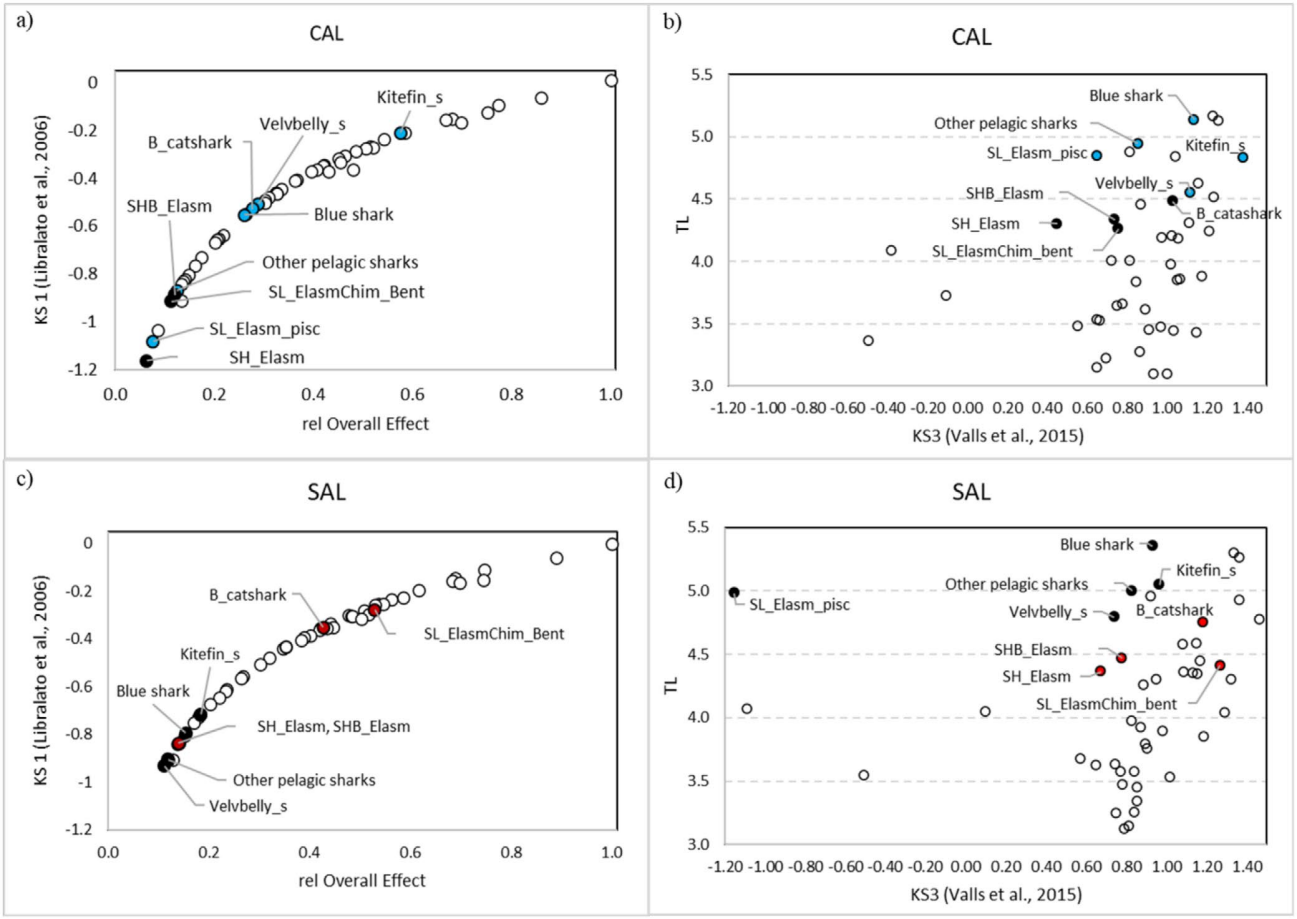
Classification of FGs in accordance with the relative Overall Effect and KS1 (panels a and c) and Trophic Level and KS3 (panels b and d) for the CAL (above) and SAL (below) food‐web models. Chondrichthyes FGs with a higher value for the KS index in one of the two webs are either in red (Salento) or blue (Calabria). Dashed lines in panels (b) and (d) indicate intervals of 0.5 in trophic level.

The position of Chondrichthyes FGs in the KS1 rank differed between the two investigated food‐web models (Figure 3a–c). In the CAL model, Kitefin_s was the most important keystone group among Chondrichthyes (9th position in KS1 rank). Velvbelly_s, B_catshark and the blue shark were other important keystone species. In the SAL food web, SL_ElasmChim_bent and B_catshark assumed the highest positions in the KS1 rank among Chondrichthyes. According to the KS3 rank, pelagic and kitefin sharks were the most important keystone predators (TL > 5.0) in the CAL food web (Figure 3b–d). Differently, elasmobranch groups of middle trophic levels assumed a more important keystone role in the SAL food web.

**FIGURE 4:**
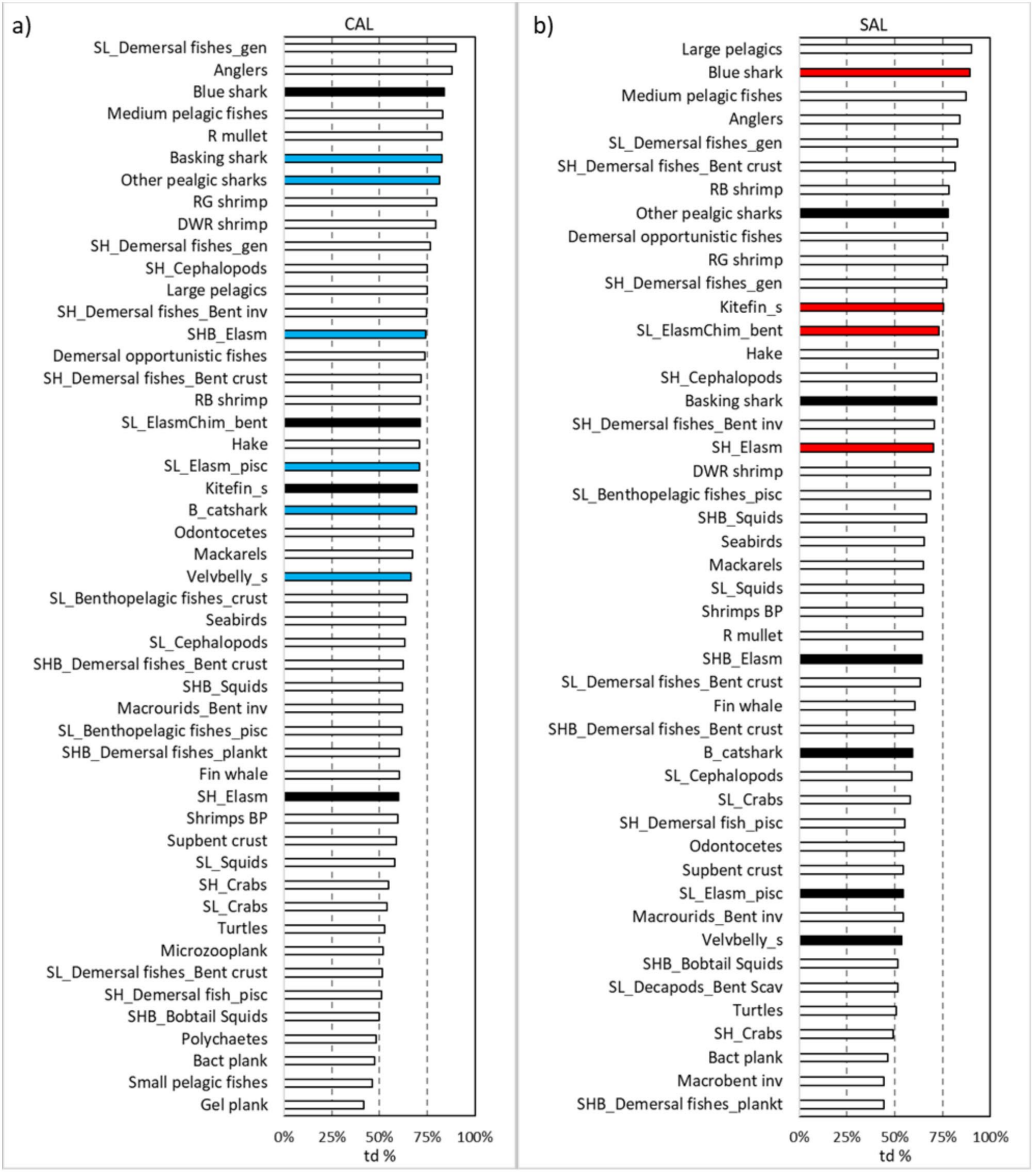
Top‐down effects (td%; > 40%) played by each FGs within (a) CAL and (b) SAL food webs. Chondrichthyes FGs with a higher value for a td% in one of the two webs are either in red (Salento) or blue (Calabria).

The highest values of top‐down effect were estimated for the blue shark in both food webs, with values of 90% and 84% in the SAL and CAL food webs, respectively (Figure 4a,b). Kitefin_s showed top‐down effects ranging between 70% and 76% between the two food webs. In the CAL food web, Velvbelly_s and B_catshark showed greater top‐down effects (> 65%) than those observed in the SAL one. The lowest td% values were detected for SH_Elasm (60%) in the CAL food web, and for Velvbelly_s (54%) in the SAL one.

**FIGURE 5:**
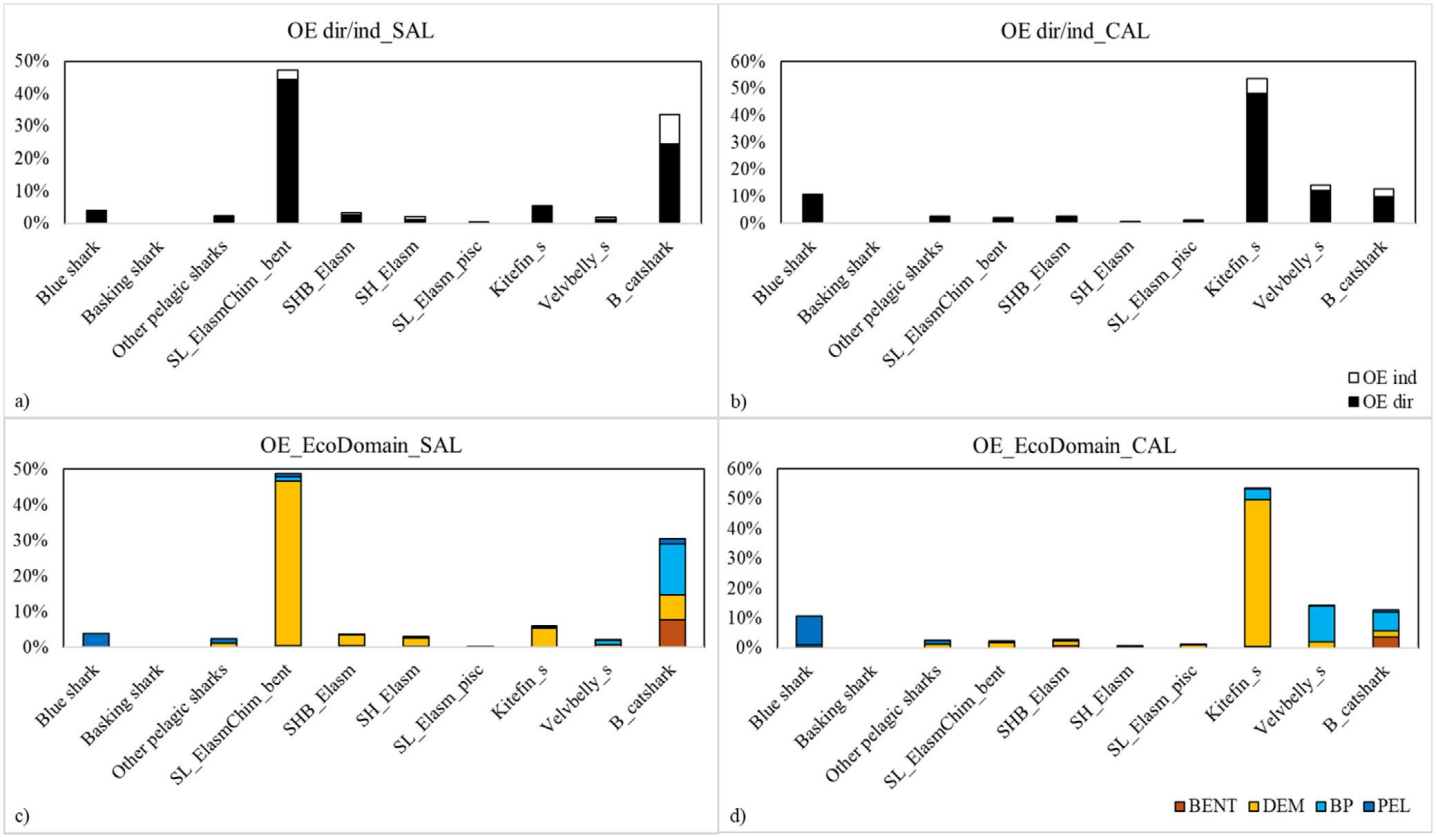
Estimations of Overall Effects (OE, expressed as %) played by Chondrichthyes FGs in the Salento and (SAL) Calabria (CAL) food webs. OE is split into direct and indirect impacts (a, b), and between the ecological domains (c, d). Ecological domains are indicated as Pelagic (PEL), Benthopelagic (BP), Demersal (DEM) and Benthic (BENT).

Chondrichthyes FGs showed direct OE higher than indirect ones in all food webs (Figure 5a,b). Kitefin_s had the most important direct OE (48% of the total impacts) in the CAL food web, followed by Velvbelly_s (12%), blue shark (11%) and B_catshark (10%). Moreover, Kitefin_s showed a small contribution to indirect OE (5%). In the SAL food web, SL_ElasmChim_bent (44%) and B_catshark (25%) showed higher contributions to direct OE. Pelagic sharks showed only direct OE in both food webs with prevailing impacts on pelagic FGs (Figure 5c,d). In the CAL food web, Kitefin_s showed OE focused on the demersal domain (49%). In the SAL food web, SL_ElasmChim_bent was characterised by OE impacting on the demersal domain (46%), while B_catshark showed higher OE values for the benthopelagic (14%) and benthic (8%) domains.

**FIGURE 6:**
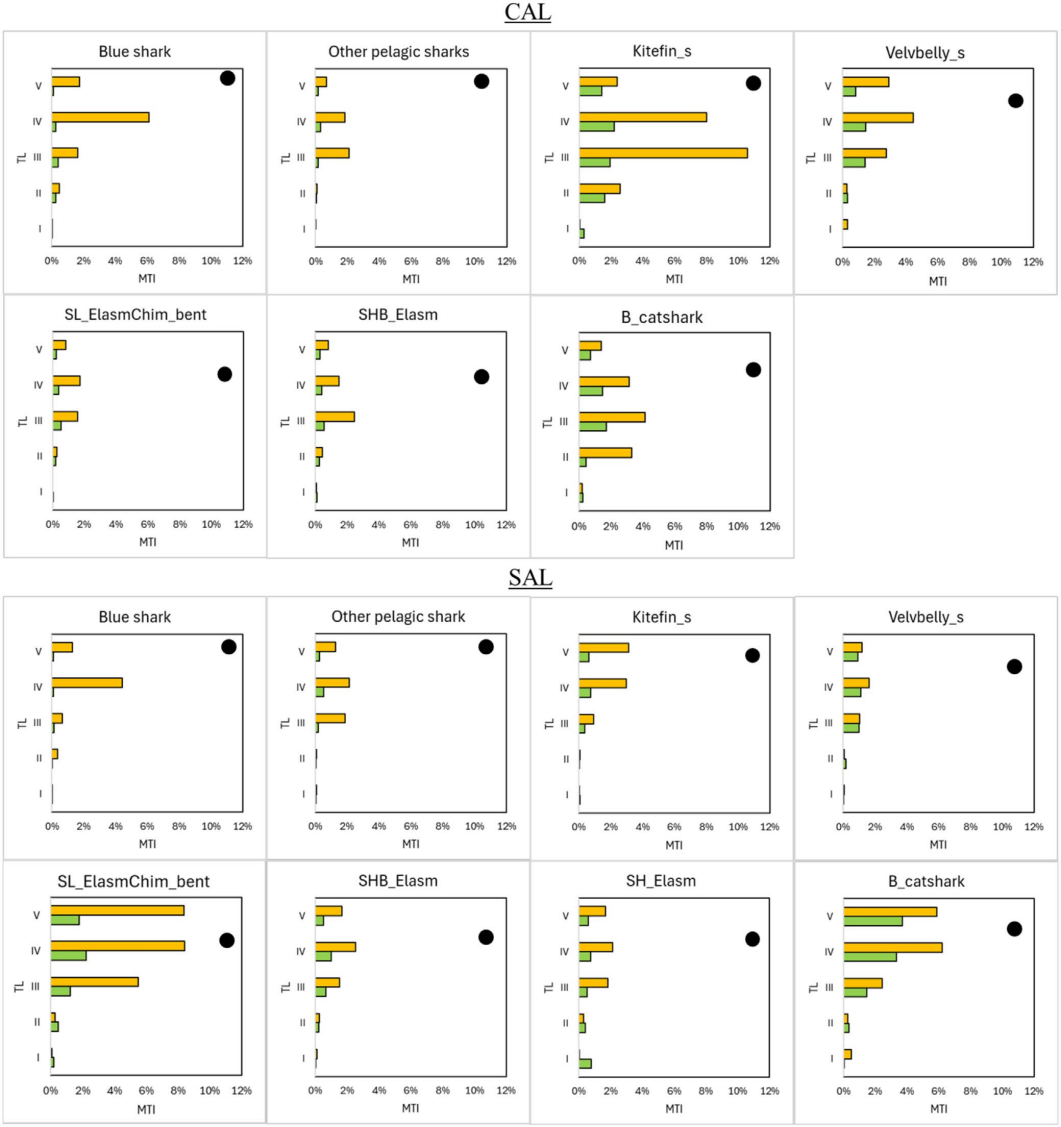
Trophic impacts played by Chondrichthyes FGs on trophic levels (*y*‐axes in roman numerals) divided into negative (orange bar) and positive (green bar) in the CAL and SAL food webs. Chondrichthyes FGs selected in the analysis were those with a contribution to the total impact greater than 5%. Trophic levels of each Chondrichthyes FG are marked by a black circle on the *y*‐axis.

All Chondrichthyes FGs showed negative impacts higher than positive ones on all discrete TLs (Figure 6). The blue shark, Kitefin_s and Velvbelly_s were the most important FGs for the top‐down controls in the CAL area. The former shark exerted exclusively top‐down controls with negative impacts on TL IV. On the contrary, other sharks showed both negative and positive impacts on all TLs, having complex indirect interactions, with the highest negative impacts on TLs III and IV, and positive impacts on TL II and I. Other pelagic sharks showed top‐down controls similar to those of the blue shark. In the SAL food web, the highest contribution to top‐down controls along TLs was detected for the B_catshark and SL_ElasmChi_bent, which both showed high negative impacts on TLs V–III, while high positive impacts were observed on TL II. A similar impact pattern was observed for the SH_Elasm, with positive impacts on TL II and I higher than negative ones.

### 3.2 Fishery Impacts on Chondrichthyes and Ecosystem Traits

The fishery exploitation estimated *F*/*Z* values greater than 0.50 for SL_Elasm_pisc and all pelagic sharks' groups (Figure 2d). Overall, the fishery exploitation showed higher levels in the CAL area than in the SAL one.

**FIGURE 7:**
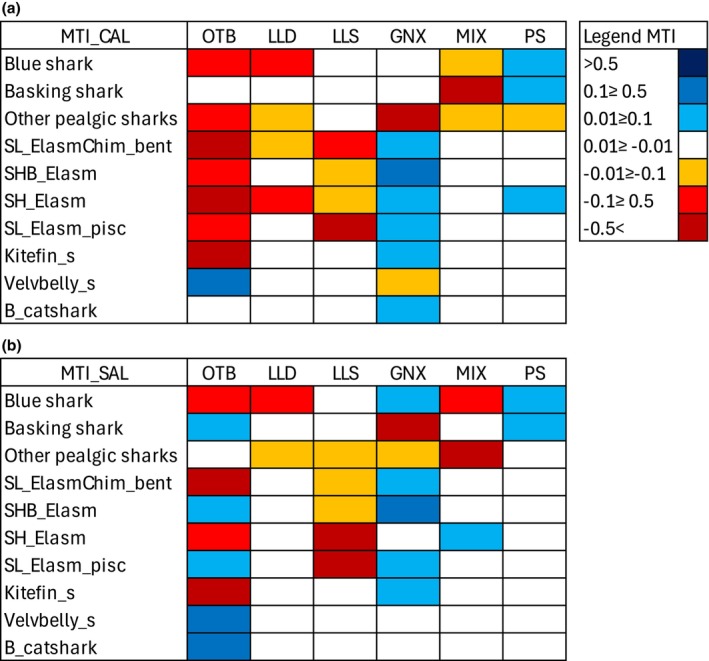
Mixed trophic impacts (MTI) by fishing gears estimated in the CAL (a) and SAL (b) models. Fishing gears (in column) are codified as: otter bottom trawl (OTB), drifting longline (LLD), setting longline (LLS), passive nets (GNX), mixed gears (MIX), and purse seine (PS).

The MTI fishing impacts played by OTB showed the highest negative values in the CAL model on all FGs, except for the Basking shark, Velvbelly_s and B_catshark (Figure 7a,b). In the SAL food web, OTB showed strong negative impacts on the blue shark, SL_ElasmChi_bent, SH_Elasm, and the Kitefin_s, while positive effects were estimated on demersal groups (Velvbelly_s, B_catshark, SHB_Elasm and SL_Elasm_pisc). LLD and LLS only showed negative impacts on pelagic and demersal Chondrichthyes, with high relevant values for the blue shark and SL_Elasm_pisc in both areas, and SH_Elasm in the SAL one. GNX showed high negative impacts on other pelagic sharks in the CAL food web and the basking shark in the SAL one. Less relevant impacts were estimated for MIX and PS. Notably, PS played positive impacts on the Basking and blue sharks in both food webs.

Ecosystem traits resulted very differently between the two areas in terms of the size of energy flows and the maturity of the systems in terms of biomass and production ratios (P/R, P/B and B/TST) (Table 3). Trophic conditions were always different over time, showing a higher primary production and a greater energy flows system in SAL than CAL, according to NPP and TST values obtained by the models. The degree of ecosystem maturity was higher in the SAL area than that of the CAL area, while no relevant differences were observed in the recycling of matter.

**TABLE 3:** Ecosystem traits estimated for Salento (SAL) and Calabria (CAL) models.

Ecosystem traits	SAL	CAL
*Q* (t km^−2^ year^−1^)	2971.2	1496.8
*E* (t km^−2^ year^−1^)	1302.3	1329.7
*R* (t km^−2^ year^−1^)	1131.4	510.3
FD (t km^−2^ year^−1^)	3686.6	3361.3
TST (t km^−2^ year^−1^)	9091.5	6698.1
*P* (t km^−2^ year^−1^)	3359.1	2316.7
NPP (t km^−2^ year^−1^)	2430.2	1329.1
PP/R	2.15	3.60
PP/BB	25.74	43.35
B/TST (year^−1^)	0.010	0.006
TB (t km^−2^)	94.4	42.4
CI	0.23	0.23
SOI	0.28	0.31
mTE (%)	28.0	22.0
FCI (% of throughput)	7.01	7.15
FPL	3.74	3.64
TC (t km^−2^ year^−1^)	1.2926	1.3410
mTLc	3.93	3.83
GE	0.0005	0.0007

*Note:* Indicator codes are reported in Appendix S1.

## 4 Discussion

The global decline in sharks and rays is a critical aspect of the structure and functioning of marine communities (Ferretti et al. 2013) and food web stability (Heithaus et al. 2008), with potential negative implications for the dynamic equilibrium of ecosystems and the provisioning of ecosystem services (Hammerschlag et al. 2019). The variety of ecological roles played by elasmobranchs in marine ecosystems has been widely proven at a global level (Dedman et al. 2024). Their importance in trophic controls can change according to the species, and ecosystem features. Results stress a high diversity in the trophic regulation exerted by pelagic and demersal elasmobranchs in two marine food webs in the Northern Ionian Sea. A detailed refinement in the description of the food webs was carried out increasing the details on two important pelagic sharks (blue and basking sharks), allowing for a more comprehensive analysis of the ecological roles and importance of these apex predators and mesoconsumers. Food‐web models were compared using the same structure in terms of FGs avoiding bias due to differences in species aggregation levels, which affect the calculation of ecological indicators (Abarca‐Arenas and Ulanowicz 2002).

### 4.1 Diversity in the Ecological Role of Pelagic and Demersal Chondrichthyes

Chondrichthyes in NIS food webs range between TL 4.26 (SL_ElasmChim_bent in CAL) and 5.35 (blue shark in SAL), excluding the basking shark that showed a TL of 3.2 (SAL). This latter value is consistent with the estimates reported in the Northeast Pacific Ocean, indicating a diet based on small and large zooplankton (Bizzarro et al. 2017). The biomass used as model input for *C. maximus* was calculated only for the SAL area, which proved to be an elective zone for the migration of this shark (Carlucci et al. 2014). Its occurrence seems to be driven by several variables affecting primary production, such as the vertical flux of particulate organic matter on the seabed (Austin et al. 2019; Finucci et al. 2021). In the Salento area, the water circulation favors particulate matter exchanges between the seabed and water surface influencing zooplankton dynamics (Mazzocchi et al. 2003), which could be favorable for the occurrence of basking sharks.

Considering other Chondrichthyes FGs, the positions occupied in both food webs and the body size highlight a general division between apex and meso‐predators. Therefore, blue, mako and thresher sharks are the most relevant apex pelagic predators together with odontocetes (Carlucci et al. 2021). Blue shark showed a TL like that estimated for the same species (5.19) in the Gulf of Lion (Seyer et al. 2023). SL_Elasm_pisc are characterised by piscivorous feeding habits and relatively larger sizes than other bathyal elasmobranchs (Barría et al. 2015). For SL_ElasmChim_bent ( *Hexanchus griseus* , *Dipturus oxyrynchus*) a high trophic level was estimated in the two food webs (4.41–4.26), as was that of *H. griseus* (4.34) in the Catalan slope model (Tecchio et al. 2013). Differently, estimations of TLs for *D*. *oxyrynchus* and *H. griseus* were higher than those obtained by stable isotope analysis (3.8–4.1) in the Northwestern Mediterranean Sea (Barría et al. 2015). SH_Elasm (*M. mustelus, R. asterias*) and SHB_Elasm (*S. canicula, Raja miraletus*) can be classified as meso‐predators (TL range = 4.3–4.5), as were those estimated for *S. canicula* (4.34) and the group of rays (4.43) in the Gulf of Lion (Seyer et al. 2023).

Overall, demersal sharks assume a greater importance as keystone species than pelagic sharks in the KS1 rank. Differently, KS3 highlights the role of the blue shark as a keystone predator. This observation is coherent with the features of two KS indices, where the former is also useful for the identification of structural roles (e.g., dominant groups), while the latter provides a better identification of keystone predators with lower biomass and higher overall effects (Valls et al. 2015). Therefore, the main evidence is a differentiation in the role played by elasmobranchs between the two food webs, where apex predators ( *D. licha* and *P. glauca* ) were found to have more impact within the Calabria food web, while mesoconsumers (e.g., the blackmouth catshark) were more important in the Salento one.

The blue and other pelagic sharks had negative impacts almost exclusively characterized by direct impacts on their prey in both areas. This trophic control indicates a strong regulation focused on several mesoconsumers of TL IV belonging to the pelagic domain, without positive cascading effects over the trophic levels. This result appears consistent with observations reported for apex‐predatory pelagic sharks in oceanic ecosystems, where biomass variations in large sharks generally induce a phenomenon of predatory pressure release on the mesoconsumers (Baum and Worm 2009). A different pattern was detected for *D. licha* (apex demersal shark), which had positive impacts on other non‐prey groups inducing a top‐down cascading effect up to the basal groups. In addition, the pattern of top‐down cascading effects with higher positive impacts on the basal groups was observed with various intensities for mesoconsumers, elasmobranchs (SH_Elasm and SHB_Elasm). Several ecological factors can affect the capability of demersal sharks to exhibit stronger top‐down cascading controls than pelagic sharks (Desbiens et al. 2021). In our analysis, the environmental differences between the two ecosystems could play a key role. Indeed, species and functional groups of the demersal domain represent key nodes in the energy exchanges between benthic and pelagic domains (Ricci et al. 2022), thus apex predators and mesoconsumers can exert more relevant trophic controls on their prey and competitors. On the contrary, species in the pelagic domain are strongly affected by the bottom‐up controls with effects on foraging species, masking the trophic control by top predators (Benoit‐Bird and McManus 2012).

### 4.2 Ecosystem Traits and Fishery Impacts

Relevant differences in the energy flows and the size of the two ecosystems were estimated, with a level of primary production higher in the Salento food web than in the Calabria one, explaining the highest TLs values estimated here. Spatial ecological differences are linked to several hydrological and geomorphological features, which affect the spatio‐temporal dynamics of the primary production, macrofaunal assemblages (Carlucci et al. 2018), and the energy exchanges between ecological domains (Ricci et al. 2022). Moreover, the modelled period corresponds to the transition from a cyclonic to an anticyclonic state of the NIS gyre in both areas, with effects on the temperature and salinity, which seem to influence the temporal and spatial distribution of the demersal Chondrichthyes in the NIS (Sion et al. 2024).

The results estimated by the model indicated a condition of overexploitation for pelagic sharks. Notably, the *F*/*Z* values of pelagic sharks indicated conditions of overexploitation, but these results should consider the setting of EE based on F rates collected from different data sources.

The MTI analysis emphasizes the negative indirect impacts of certain fishing gears that compete for trophic resources with pelagic elasmobranchs, such as bottom trawl, or conversely, positive effects due to the potential removal of competitors, such as purse seine catching large pelagics. In addition, differences in MTI fishing impacts were evident between the two areas, with the highest negative ones estimated in the Calabrian area. These differences are mainly linked to a different fishing pressure in the two areas (Sion et al. 2024), and the very narrow shelf area in the Calabrian region, which increases the negative impacts on commercial elasmobranchs (Ricci et al. 2021). Drifting longlines were found to be the most negatively impacting gears on the blue and other pelagic sharks, as estimated for the blue shark in the Gulf of Lion using MTI analysis (Seyer et al. 2023).

The fishing gears' impacts identified by our analysis stress the need to implement strategies to manage fisheries and bycatch of demersal and pelagic sharks by trawl and longlines. The structural differences of these two gears should be considered in different technical actions, promoting the adoption of bycatch‐reducing devices for the trawl fishery (De Santis et al. 2024), and changes in the fishing behaviour of pelagic longlines. This latter could be an interesting action in the Salento area, considering the daily vertical movement patterns of blue sharks observed in the nearby area of the Southern Adriatic Sea (Carbonara et al. 2023). Reversing the night‐day fishing period could be a possible expedient to reduce the overlap between the presence of blue sharks at the surface during the night and fishing activities. Finally, the identified high impacts of passive nets on basking sharks require urgent investigations to better understand the migratory dynamics of this species and the overlap with potential fishing areas in a scenario of relevant climatic variations that may affect the spatial distribution of this species (Sun et al. 2024).

### 4.3 Insights Into Modelling Assumptions for Pelagic Sharks and Differences in Food‐Web Models

Pelagic predator sharks are often considered in this kind of model as apex predators without predation by other top consumers. However, juvenile pelagic sharks could be affected by low predation mortality due to hunting events by large odontocetes (Mucientes and González‐Pestana 2020), such as sperm whales on several pelagic sharks (Gaskin and Cawthorn 1967). The latter species is regularly present in the Ionian area (Carlucci et al. 2021), and therefore, it could be considered a potential predator of the blue shark. At the same time, the direct predation of large pelagic sharks on small odontocetes has not been represented in this model due to the absence of direct local evidence; although, the phenomenon has been documented for the mako shark in the Atlantic area (Monteiro et al. 2006; Biton‐Porsmoguer et al. 2015). Thus, the realism of the model could be improved in the future, with new diet information and representing migration movements for some pelagic sharks, such as the blue shark population distributed between the Adriatic and Ionian regions (Carbonara et al. 2024).

The approach adopted in the building of the food webs structure in the models allows an unbiased comparison between the investigated food webs, since the use of the same FGs avoids the treatment of data to standardise the modelled trophic structure (Libralato et al. 2010). Overall, the two models are characterised by a high quality of data relative to the groups belonging to the demersal, pelagic and benthic domains and placed in the middle and high TLs. On the other hand, simplifications occurred for the groups of plankton and benthic invertebrates (excluding the decapod crustaceans) in both food‐web models, something not uncommon in Ecopath models, so that biomass data were estimated fixing EE values. The lower data accuracy of plankton groups and macrobenthic invertebrates could mainly affect the estimates of ecosystem‐level traits in both models, where the contribution of the basal components of the food web is essential for energy flow dynamics (Heymans et al. 2016). On the other hand, estimates obtained by the models are consistent with the ecological differences of the two areas due to several hydrological and geomorphological drivers (see Ricci et al. 2022).

In our opinion, the main limitations are represented by the lack of information on biomass data for several pelagic sharks, with the only exception for the basking sharks in the Salento area. In this case, biomass data were estimated through the availability of official catch data and the mortality components. This issue is a common case in the mass‐balance approach adopted to describe large pelagic species (e.g., Seyer et al. 2023). In the lack of biomass data, acquiring different estimates of fishing mortality data from different sources could improve the estimates of final biomasses, allowing the calculation of uncertainty ranges. For instance, some information on the fishing mortality of blue sharks by pelagic longlines was indirectly estimated by Catch Per Unit Effort values obtained from an experiment of bycatch mitigation conducted in the Central Adriatic Sea during the summer 2021 (Carbonara et al. 2023). Although this type of experimental data does not coincide with the period described by NIS models, it could nevertheless be useful for calibrating future simulation scenarios aimed at testing the reduction of elasmobranch bycatch, promoting sustainable fisheries management (Ricci et al. 2023).

Overall, this study highlights the high diversity in the trophic roles of pelagic and demersal elasmobranchs, stressing their importance in the control of meso‐predators by pelagic species and the regulation of top‐down cascading effects by demersal apex sharks. The high degree of detail of the models adopted makes it possible to identify trophic controls not only along trophic levels, but also between ecological domains, highlighting important effects on the trophic groups of the demersal and benthopelagic domains. Beyond the meal provided to a predator by its prey, indirect trophic interactions between different elasmobranchs support the ecological stability of food webs. Fishing impacts on these species lead to a weakening or loss of important ecological roles, requiring the implementation of modeling scenarios to test conservation and fishery management measures.

## Author Contributions

**F. P. De Luca:** formal analysis (equal), methodology (equal), visualization (equal). **G. Cipriano:** data curation (equal), investigation (equal), methodology (equal), writing – review and editing (equal). **R. Carlucci:** investigation (equal), resources (equal), supervision (equal), visualization (equal), writing – review and editing (equal). **L. Finotto:** conceptualization (equal), data curation (equal), methodology (equal), validation (equal), writing – original draft (equal). **C. Mazzoldi:** validation (equal), visualization (equal), writing – review and editing (equal). **C. Zampieri:** conceptualization (equal), data curation (equal), formal analysis (equal), validation (equal), visualization (equal), writing – review and editing (equal). **A. Barausse:** conceptualization (equal), formal analysis (equal), methodology (equal), validation (equal), visualization (equal), writing – review and editing (equal). **P. Ricci:** conceptualization (equal), formal analysis (equal), investigation (equal), methodology (equal), software (equal), validation (equal), writing – original draft (equal), writing – review and editing (equal).

## Conflicts of Interest

The authors declare no conflicts of interest.

## Supporting information


**Appendix S1:** Supporting Information.

**Table S1–S5:** ece372540‐sup‐0002‐TableS1–S5.xlsx.

## Data Availability

All data are available in the main text and Supporting Information (Appendix S1 and Tables S1–S5).
