# Supplementary material for: Beyond the Meal: Trophic Controls by Pelagic and Demersal Chondrichthyes in Two Different Mediterranean Marine Food Webs
Source: Ecol Evol. 2025 Nov 19;15(11):e72540. doi: 10.1002/ece3.72540 (PMC12628014; doi:10.1002/ece3.72540)
Supplement: Supplementary file 1 — Appendix S1: Supporting Information. [file ECE3-15-e72540-s001.docx]

Appendix A

Beyond the meal: trophic controls by pelagic and demersal Chondrichthyes in two different Mediterranean marine food webs

Summary

[Input data of pelagic sharks 2](#_Toc211763305)

[Other FGs and fishing gears: input data and parametrization 6](#_Toc211763306)

[Balancing models procedures 7](#_Toc211763307)

[References 9](#_Toc211763308)

## Input data of pelagic sharks

Estimating biomass is very difficult for highly mobile and migratory organisms, such as pelagic sharks. A final biomass input was only obtained for the Basking shark (C*etorhinus maximus*), while the EE for the other pelagic shark groups was fixed to estimate the biomass using the model. The occurrence of basking sharks around the Apulian coasts of the Salento area (SAL) is widely documented (De Sabata et al., 2013; Carlucci et al., 2014), while the occurrence in the Calabrian Ionian area was scarcer than those reported in the Apulian region and Tyrrhenian Sea (Sperone et al., 2012; Mancusi et al., 2020). Sightings of *C. maximus* are reported in the period 2011-2014, with estimates of the total length (L, in cm) of individuals, for the Southern Adriatic and Northern Ionian Seas. For biomass estimation, only sightings reported for the Northen Ionian Sea were considered in the calculation for the years 2011 and 2013 (Tab. A1). This choice was due to the opportunistic nature of the data, derived from reports of animals in citizen science contexts, with absence of sightings in 2012 and only one in 2014 (Carlucci et al., 2014). The L of 14 individuals (5 in 2011, and 9 in 2013) were used to obtained body mass using the length-weight relationship (W=0.00017×L^2.436^) of the basking sharks in the Mediterranean Sea (Mancusi et al., 2020). Biomasses obtained in this way were summed up within the single year, averaged between the two years considered, and standardized for the SAL study area to obtain the final biomass input. The biomass of the basking shark in the CAL model was estimated by fixing the EE (0.61), which was obtained from the balancing model of the SAL area.

Tab A1: Data of Abundance (N), Total Length (L), Distance from the coast (DC, in NM) and Depth reported in Carlucci et al.; 2013). Body mass (BM, kg) was estimated using the length-weight relationship reported in Mancusi et al. (2020).

| **Year** | **L (cm)** | **DC (NM)** | **Depth (m)** | **BM (kg)** |
| --- | --- | --- | --- | --- |
| 2011 | 500 | 0.11 | 20 | 636 |
| 2011 | 800 | 3.00 | 160 | 2000 |
| 2011 | 600 | 0.15 | 25 | 992 |
| 2011 | 600 | 2.50 | 32 | 992 |
| 2011 | 600 | 1.00 | 40 | 992 |
| 2013 | 400 | 0.15 | 5 | 370 |
| 2013 | 800 | 1.00 | 25 | 2000 |
| 2013 | 700 | 2.5 | 30 | 1444 |
| 2013 | 800 | 3.00 | 30 | 2000 |
| 2013 | 800 | 3.00 | 30 | 2000 |
| 2013 | 700 | 3.00 | 30 | 1444 |
| 2013 | 700 | 3.00 | 30 | 1444 |
| 2013 | 750 | 1.00 | 15 | 1709 |
| 2013 | 700 | 0.50 | 30 | 1444 |

For the groups of blue and other pelagic sharks, final biomass values were estimated by setting the EE in both models, with values of 0.530 and 0.648 for blue and other pelagic sharks, respectively. These values were estimated assuming that these three sharks are not affected by predation mortality being apex predators in the food web. Thus, fishing mortality (F) can be used to represent EE, which is the proportion of the production of each species consumed in the system (Christensen and Walter, 2004). Estimates of fishing mortalities are lacking for these pelagic predators in the Ionian Sea, and data of F represented as Catch Per Unit Effort (CPUE) are very dated, such as values collected in the Eastern Ionian Sea during the period 1998-2001 for blue, mako and thresher sharks using several fishing gears (Megalofonou et al., 2005). The mean CPUE calculated from this study indicated a value of 0.217 for the blue shark, while CPUE values calculated for mako and thresher sharks were extremely low and unrealistic (>0.02). Some information on the blue shark CPUE is reported from an experiment on bycatch mitigation conducted during summer 2021 with longlines in the Central Adriatic Sea (Carbonara et al., 2023). In this case, values estimated ranged between 2.78-11.11. On the contrary, several estimations of fishing morality with different methods (CPUE, satellite tagging, assessment of post-release mortality) are available in other global regions, such as the Pacific (Herber et al., 2010; Sepulveda et al., 2015; Teo et al., 2018) and the Atlantic (Campana et al., 2015; Byrne et al., 2017). In order provide input data to the models with a lower degree of uncertainty coming from a single source, F values of modelled pelagic sharks were acquired from estimates provided by a random-effect meta-analysis on post-release fishing mortality data at global level (Musyl and Gilman, 2019) (Table 2 in the main text).

*Production rate (P/B)*

The P/B rate was estimated using the empirical relationship based on the calculation of the total mortality rate (Z), which is equal to P/B in most ecological conditions in dynamic equilibrium (Allen, 1971). Thus, the Z of a population is the sum of F and natural mortality (M), and these components can be estimated through empirical equations or data available in the scientific literature (e.g., stock assessment). In particular, growth parameters from the Von Bertalanffy growth equation (K, the growth rate, and L_inf_, the asymptotic length) are required in the estimation of M.

No estimates of P/B existed for these species in the two study areas. However, it can be assumed that P/B is equal to the total mortality rate (Z) of the populations according to Allen (1971), which is the sum of fishery mortality (F) and natural mortality (M). Therefore, P/B could be calculated from these components through empirical equations or data available in the scientific literature. As no published values of M existed for the study areas, it was estimated according to the equation of Pauly (1980):

${Log}_{10}M=0.654{log}_{10}K-0.28{log}_{10}L_{inf}+0.463{log}_{10}T$ (eq. A1)

where K (the annual growth rate), L_inf_ (the asymptotic length expressed in cm) are parameters estimated from the Von Bertalanffy growth equation, and T is the mean temperature of the water (in °C) recorded for the period in which the species occurs in the study area. Growth parameters were sourced from published literature for areas as close as possible to the study area: Mediterranean Sea (Adriatic, Ionian, Aegean, and Levantine Seas) for blue sharks (Megalofonou et al. 2009); Central Pacific Ocean for thresher sharks (Gervelis and Natanson, 2013); Western and Central Atlantic Oceans for mako sharks (Barreto et al., 2016); and North-Eastern Atlantic Ocean for basking sharks (Pauly, 2002) (for more details, see Table 2). In the Northern Ionian Sea, the average sea surface temperature recorded during the months with the highest basking shark occurrence (January–April, Carlucci et al., 2014) was 14.57 °C. For the other shark species, the yearly sea surface temperature in the Northern Ionian Sea was used (19° C), averaging winter and summer values sourced from an online database (https://seatemperature.info/april/ionian-sea-water-temperature.html). For blue, mako, and thresher sharks, F estimates were acquired from scientific studies reported in Musyl and Gilman (2019), and then F and M values were summed for the final calculation of total mortality rate (M+F=Z=P/B). For basking sharks, estimates of P/B rates were not available for the SAL area. However, assuming a direct proportion between Z and M, total mortality rate was calculated using Z and M estimated for the North-Eastern Atlantic (Pauly, 2002) and M calculated for the study area. This estimate might be slightly inflated because values reported by Pauly (2002) partially include the effects of target fisheries existing in the area.

*Consumption rate (Q/B)*

The Q/B rate for the common thresher shark was empirically calculated from the relationship reported in Palomares and Pauly (1999):

${Log}_{10}Q/B=7.964-0.204 {log}_{10}W_{inf}-1.965 T^{'}+0.083 A+0.532 h+0.398 d$ (eq. A2)

where W_inf_ is the asymptotic weight (i.e. the maximum weight an animal would reach if it could grow indefinitely; in g), T' is the temperature of the study area in degree Kelvin, A is the aspect ratio of the caudal fin, and *h* and *d* are dummy variables assuming different values for herbivores (*h* = 1, *d* = 0), carnivores (*h* = 0, *d* = 0), and detritivore animals (*h* = 0, *d* = 1). W_inf_ was calculated by averaging L_inf_ between sexes and converting it to weight according to the length-weight relationship estimated for the population of the North Atlantic Ocean (Gervelis and Natanson, 2013). The value for A (2.91) was obtained from direct measurements of 4 tail pictures collected within a study addressed to analysis the relationships between habitat traits and the caudal fin morphometry (unstill unpublished data, Bressan et al., submitted). Temperature expressed in degree Celsius (T°C), the same used for P/B calculations, was converted to Kelvin following the equation:

$T^{'}=\frac{1000K}{\left( T^{\circ}C+273.15 \right)}$ (eq. A3)

For the other pelagic sharks, a reformulation of the previous equation allowed the conversion of published Q/B values (Q/B_T1_), estimated for different areas and temperatures (T1), to obtain a value for the Northern Ionian Sea (Q/B_T2_) according to the temperature of the study area (T2).

${Q/B}_{T2}={Q/B}_{T1}\times\left( {{{10}^{-1.965*T2}}/{10}}^{-1.965*T1} \right)$ (eq. A4)

Temperature values for the Northern Ionian Sea (T2) were the same as those adopted for the calculation of P/B rates. For basking shark, Q/B conversions were based on values estimated for an animal of an average total length of 5-7 m, inhabiting the English Channel at a temperature of 13.35 °C (Sims, 2008). For the blue shark, Q/B was calculated from bioenergetic models of animals living in the Central Pacific Ocean at an average temperature of 16.0 °C (Kitchell et al., 2022). For the mako shark, the original Q/B value was sourced from animals distributed in the South Atlantic Ocean at 23.75 °C (Bornatowski et al., 2018). Final P/B and Q/B values for the Other pelagic sharks (FG 8) were calculated by using the average of those estimated for the thresher and mako sharks

Diet

Quantitative data on the diet of pelagic sharks were acquired from studies based on the stomach content analysis for carnivorous species, while the diet of the basking shark was obtained from a study on the planktonic community sampled during the active feeding of *C. maximus* in southwest England waters (Sims and Merrett, 1997). Diet information for the blue shark was obtained from the Ligurian Sea (Garibaldi and Orsi Relini, 2007), that for thresher shark from the California marine ecosystem (Pacific Ocean; Preti et al., 2012), and that for mako shark from the Northeast Atlantic Ocean, off Portugal (Maia et al., 2004; Biton-Porsmoguer et al., 2017). Diets of blue and mako sharks were adjusted excluding seabirds and odontocetes as their presence in sharks’ stomach content was probably due to opportunistic feeding on discarded/dead animals rather than from active predation. The diets of pelagic and demersal Chondrichthyes were included with the diets of other groups collected in previous local models (Carlucci et al., 2021; Ricci et al., 2019; 2021) and were subsequently aggregated into a predator-prey matrix by FG to describe trophic interactions in both models (Suppl. Mat., Tab. S3).

## Other FGs and fishing gears: input data and parametrization

Biomasses of pelagic species (Odontocetes, Fin whale, Loggerhead turtle) were calculated through abundance data (N km^-2^) multiplied for the mean individual weight of the species included in these groups. Mean individual weight for odontocetes (striped dolphin, common bottlenose dolphin Risso’s dolphin) were acquired from local estimations based on stranding data (Carlucci et al., 2024). For the fin whale and the Loggerhead turtle, abundance data were acquired from OBIS SeaMap (Halpin et al., 2009) and individual mean weights were adopted from previous local models (Carlucci et al., 2021). Biomass data not available for benthic and planktonic groups were estimated by the model (Christensen et al., 2008), fixing the EE for the Polychaetes, Macrobent inv and Gel plank (EE=0.95), Supbent crust, Macrozooplank, Mesozooplank, Microzooplank (EE=0.99), Macrophyto benthos (EE=0.75), Large phytoplankton (EE=0.50), and Small phytoplankton (EE=0.30). Bacterioplankton biomasses were acquired from local available models for the NIS (Carlucci et al., 2021). P/B and Q/B rates acquired from local models of the Salento area and the Gulf of Taranto (Carlucci et al., 2021).

New updated official fishery data (landings and bycatch, in annual kg) acquired from the Italian National Fisheries and Aquaculture Economic Research Institute (NISEA), were used to represent the local fishing gears: otter bottom trawl (OTB), drifting longline (LLD), setting longline (LLS), passive nets (GNX), mixed gears (MIX), and purse seine (PS) in both models. Data were obtained as Ionian Apulian and Calabria subregions for the period 2005-2021. In the case of missing data for a given species during the modelled period (2013-2015), the average value of the time series was applied. Data were successively standardised for the surface of each modelled area in t km^-2^ and summed in the corresponding FG (Table S4). Discards derived from commercial *taxa* were calculated using discard rates by gears and *taxa* acquired from several data sources (Tsagarakis et al., 2014; Sartor et al., 2016). The discard fraction for non-commercial *taxa* caught by OTB were estimated based on the proportion of commercial and non-commercial discards in MEDITS data for the period of investigation (Ricci et al., 2019).

## Balancing models procedures

The balancing of both models was conducted by applying the standard procedure based on the check and adjustment of input data coherently with basic thermodynamic laws, as well as the rules and principles of ecosystem ecology at the system level (Heymans et al., 2016). In a first step, FGs were balanced by adopting a top-down approach (Mackinson and Daskalov, 2007), checking the expected limit of EE (must be <1) (Christensen et al., 2008). A total of 11 and 4 FGs showed an EE>1 in the SAL and CAL models, respectively, indicating that these groups were characterized by over-predation-fishery in the system. In the case of excessive fishing mortality for these FGs (e.g. the case of groups in the CAL model, in which new input data concern landings and discards), the correction of EE was carried out by revising the values of discard rates for non-commercial species. The reduction in high predation mortalities on the groups was carried out by adjusting the values in the diet matrices, starting from those diets data from areas far from the modelled areas. The modification of diet matrices is preferred to the adjustment of other parameters (B, P/B and Q/B), which are in most cases calculated on the basis of local sampling data and empirical equations. The high EE for the Large pelagics group (tuna and swordfish) due to the high catches in both models being balanced by adding an immigration rate (0.070 and 0.025 t km^-2^ y^-1^ in the SAL and CAL models, respectively), which represents the input of biomass in the modelled area due to the migratory behaviour of this species (Agnetta et al., 2019). After the balancing of all EE values, both models were evaluated though a pre-balancing analysis (PREBAL, Link, 2010) to assess whether data were coherent with some basic ecological rules. The following diagnostics were checked: biomass across taxa/trophic levels (where biomass should span 5–7 orders of magnitude and slope on log scale should be a 5–10% decline), vital rates across taxa/trophic levels (should be a general decline with increasing trophic level), the net food conversion efficiencies as P/Q rate (0.05-0.3 usually for fish groups and <0.5 for planktonic groups), and the Respiration/Assimilation rate (must be <1) (Heymans et al., 2016).

The quality of input data in both models was calculated by categorizing the different input sources through the Pedigree routine, which provide an index ranged between 0 (low quality) and 1 (high quality). The Pedigree index was equal to 0.65 for both models, suggesting the acceptable quality of the models.

Table A2. Ecosystem and Fishing indicators (Ind.) adopted in the description of ecosystem traits.

| **Ind.** | **Name** | **Definition** |
| --- | --- | --- |
| Q | Consumption flows (t km^-2^ y^-1^) | Total consumption flows in the food web |
| E | Export flows (t km^-2^ y^-1^) | Total export flows from the food web |
| R | Respiration flows (t km^-2^ y^-1^) | Total Respiration flows in the food web |
| FD | Detritus flows (t km^-2^ y^-1^) | Total flows to the detritus |
| TST | Total System Throughput (t km^-2^ y^-1^) | The sum of all flows in a system, representing the ‘size of the entire system. |
| P | Sum of all Production (t km^-2^ y^-1^) | Sum of all production in the food web |
| NPP | Net Primary Production (t km^-2^ y^-1^) | NPP is calculated as the summed primary production from all producers. |
| TB | Total Biomass (t km^-2^ y^-1^) | Total biomass excluding all detritus functional groups |
| PP/R | Total Primary Production/total Respiration | The ratio describes the maturity of an ecosystem. Immature system PP/R >1; Mature system PP/R ~1; Stressed system PP/R <1. |
| PP/B | Total Primary Production/total Biomass | The ratio is expected to be a function of the system’s maturity. Early development stage PP/B >>1; mature system PP/B ~ 1. |
| B/TST | Total Biomass/Total System Throughputs (y-1) | The ratio describes the ecosystem maturity, and it can take any positive value. Increasing values indicate mature stages of an ecosystem. |
| CI | Connectance Index | The ratio of the number of actual links to the number of possible links: CI = N (N-1)^2^. High CI values indicate high complexity and maturity of the system. |
| SOI | System Omnivory index | The average omnivory index of all consumers weighted by the logarithm of each consumer's food intake. SOI is a measure of how the feeding interactions are distributed between trophic levels. An omnivory index is also calculated for each consumer FG, indicating the variance of the TL estimate for the FG. |
| FCI | Finn’s Cycling Index (%) | Fraction of amount flows cycled over the total of an ecosystem’s throughput. FPL is an index of flows recycling, where high values indicate a mature system. |
| FPL | Finn’s mean Path Length | FPL is defined as the average number of groups that an inflow or outflow passes through. High values indicate a mature system. |
| mTE | mean Transfer Efficiency (%) | Geometric mean of transfer efficiencies for trophic level II to IV. |
| TC | Total Catches (t km^-2^ y^-1^) | Total landings and discards exported from the system. |
| mTLc | mean Trophic Level of catches | Average trophic level of all caught species weighted by yield. |
| GE | Gross Efficiency | GE is the ratio between TC/NPP, which measures the efficiency of a fishery. Values are generally lower than 1 and lower values indicate a low efficiency. |

## References

Agnetta, D., Badalamenti, F., Colloca, F., *et al.* (2019). Benthic‐pelagic coupling mediates interactions in Mediterranean mixed fisheries: An ecosystem modeling approach. *PLoS ONE*, 14(1), e0210659. https://doi.org/10.1371/journal.pone.0210659

Allen, R.R. (1971). Relation Between Production and Biomass. *J. Fish. Res. Board Can.*, 28, 1573–1581. doi: 10.1139/f71-236

Barreto, R.R., de Farias, W.K.T., Andrade, H., Santana, F.M., Lessa, R., (2016). Age, growth and spatial distribution of the life stages of the shortfin mako, *Isurus oxyrinchus* (Rafinesque, 1810) caught in the Western and Central Atlantic. *PLoS ONE*, 11 (4): e0153062. <https://doi.org/10.1371/journal.pone.0153062>

Biton-Porsmoguer, S.B., Bănaru, D., Boudouresque, C.-F., Dekeyser I., Béarez, P., Lozano, R.M. (2017). Compared diet of two pelagic shark species in the northeastern Atlantic Ocean. *V&M*, 67(1): 21-25.

Bornatowski, H., Braga, R.R., Barreto, R.P. (2018). Elasmobranchs consumption in Brazil: impacts and consequences. Rossi-Santos MR, Finkl CW (Eds) Advances in marine vertebrate research in Latin America. Springer, 251–262. https://doi.org/10.1007/978-3-319-56985-7_10

Byrne, M.E., Cortés, E., Vaudo J.J., Harvey G.C. McN., Sampson, M., Wetherbee, B.M. Shivji, M. (2017). Satellite telemetry reveals higher fishing mortality rates than previously estimated, suggesting overfishing of an apex marine predator. *Proc. R. Soc. B.*, 28420170658http://doi.org/10.1098/rspb.2017.0658

Campana, S.E., Fowler, M., Houlihan, D., Joyce, W., Showell, M., Miri, C., Simpson, M. (2015). Current status and threats to the North Atlantic Blue Shark (*Prionace glauca*) population in Atlantic Canada. DFO Can. Sci. Advis. Sec. Res. Doc. 2015/026

Carbonara, P., Prato, G., Niedermüller, S., *et al.* (2023). Mitigating effects on target and by-catch species fished by drifting longlines using circle hooks in the South Adriatic Sea (Central Mediterranean). *Front Mar Sci*, 10:1124093. https://doi.org/10.3389/fmars.2023.1124093

Carlucci, R., Battista, D., Capezzuto, F., Serena, F., Sion, L. (2014). Occurrence of the basking shark *Cetorhinus maximus* (Gunnerus, 1765) (Lamniformes: Cetorninidae) in the central-eastern Mediterranean Sea. *Ital. J. Zool.*, 81:2, 280-286. https://doi.org/10.1080/11250003.2014.910275

Carlucci, R., Capezzuto, F., Cipriano, G., *et al.* (2021). Assessment of cetacean–fishery interactions in the marine food web of the Gulf of Taranto (Northern Ionian Sea, Central Mediterranean Sea). *Rev. Fish Biol. Fish*., 31, 135–156. https://doi.org/ 10.1007/s11160-020-09623-x

Carlucci, R., Ricci, P., Ingrosso, M., Cascione, D., Fanizza, C., Cipriano, G. (2024) Estimates of length-weight relationships and consumption rates of odontocetes in the Mediterranean Sea from stranding data. *Estuarine, Coastal Shelf Sci.*, 298, 108622. https://doi.org/10.1016/j.ecss.2024.108622

Christensen, V., Walters, C., Pauly, D., Forrest R., (2008). Ecopath With Ecosim 6: A User’s guide. Fisheries Centre University of British Columbia, Vancouver, BC (2008)

Christensen, V., and Walters, R. (2004). Ecopath With Ecosim: methods,capabilities and limitations. *Ecol. Modell.*, 172 (2–4), 109–139. doi: 10.1016/j.ecolmodel.2003.09.003

De Sabata, E., Bello, G., Cataldini, G., Mancusi, C., Serena, F., Clò, S. (2013). A seasonal hotspot for *Cetorhinus maximus* in Apulia, Southern Italy, Mediterranean Sea. EEA 17th Annual Scientific Conference, 1-3 November 2013, Plymouth, England.

Garibaldi, F., Orsi Relini, L. (2000). Summer abundance, size and feeding habits of the blue shark, *Prionace glauca*, in the pelagic sanctuary of the Ligurian Sea. *Biol. mar. mediterr.*, 7(1): 324 – 333.

Gervelis, B.J., and Natanson, L.J. (2013). Age and growth of the thresher shark, *Alopias vulpinus*, in the western north Atlantic Ocean. *Trans. Am. Fish. Soc.*, 142: 1535-1545

Halpin, P.N., Read, A.J., Fujioka, E., *et al.* (2009). OBIS-SEAMAP: the world data center for marine mammal, sea bird and sea turtle distributions. *Oceanography*, 22 (2), 104-115.

Heberer, C., Aalbers, S.A., Bernal, D., Kohin, S., DiFiore, B., Sepulveda, C.A. (2010). Insights into catch-and-release survivorship and stress-induced blood biochemistry of common thresher sharks (*Alopias vulpinus*) captured in the southern California recreational fishery. *Fish. Res.*, 106, 495–500. https://doi.org/10.1016/j.fishres.2010.09.024

Heymans, J.J., Coll, M., Link, J.S., *et al.* (2016). Best practice in Ecopath with Ecosim food-web models for ecosystem-based management. *Ecol. Modell.*, 331, 173–184.

Kitchell, J.F., Essington, T.E., Boggs, C.H., Schindler, D.E., Walters, C.J. (2022). The role of sharks and longline fisheries in a pelagic ecosystem of the central pacific. *Ecosystems*, 5:202-216. https://doi.org/10.1007/s10021-001-0065-5

Link, J. S. (2010). Adding rigor to ecological network models by evaluating a set of pre‐balance diagnostics: A plea for PREBAL. *Ecol. Modell.*, 221, 1582–1593.

Mackinson, S., & Daskalov, G. (2007). An ecosystem model of the North Sea for use in research supporting the ecosystem approach to fisheries management: Description and parameterisation. Cefas Science Series Technical Report, pp. 142.

Maia, A., Queiroz, N., Correia, J.P., Cabral, H. (2006). Food habits of the shortfin mako, Isurus oxyrinchus, off the southwest coast of Portugal. *Env. Biol. Fishes*, 77, 157–167.

Mancusi, C., Baino, R., Fortuna, C., *et al.* (2020). MEDLEM database, a data collection on large Elasmobranchs in the Mediterranean and Black seas. *Mediterr. Mar. Sci.*, 21, 276-288.

Megalofonou, P., Damalas, D., Deflorio, M., De Metrio, G. (2009). Modeling environmental, spatial, temporal, and operational effects on blue sharks by-catches in the Mediterranean long-line fishery. *J. Appl. Ichthyol.*, 25(1), 47-55. https://doi.org/10.1111/j.1439-0426.2009.01221.x

Megalofonou, P., Damalas, D., Yannopoulos, C. (2005). Composition and Abundance of Pelagic Shark by-Catch in the Eastern Mediterranean Sea. *Cybium*, 29: 135-140. https://doi.org/10.26028/cybium/2005-292-004

Musyl, M.K., Gilman, E.L. (2019). Meta-analysis of post-release fishing mortality in apex predatory pelagic sharks and white marlin. *Fish. Fish*., 20, 466–500. 10.1111/faf.12358

Palomares, M.L.D., and Pauly, D. (1999). Predicting the food consumption of fish populations as functions of mortality, food type, morphometrics, temperature and salinity. *Mar. Freshw. Res.*, 49, 447-453

Pauly, D. (1980). On the interrelationships between natural mortality, growth parameters, and mean environmental temperature in 175 fish stocks. *ICES J. Mar. Sci.*, 39(2), 175–192. https://doi.org/10.1093/icesjms/39.2.175.

Pauly, D. (2002). Growth and mortality of the basking shark *Cetorhinus maximus* and their implications for management of whale *Rhincodon typus*. p. 199-208. In S.L. Fowler, T. Reid and F.A. Dipper (eds.). Elasmobranch Biodiversity, Conservation and Management: Proceedings of the International Seminar and Workshop, Sabah, Malaysia, July 1997. Occasional Papers of the IUCN Survival Commission No. 25, Gland, Switzerland.

Preti, A., Soykan, C.U., Dewar, H., Wells, R.J.D., Spear, N., Kohin, S. (2012). Comparative feeding ecology of shortfin mako, blue and thresher sharks in the California Current. *Environ. Biol. Fishes*, 95, 127–146. https://doi.org/10.1007/s10641-012-9980-x

Ricci, P., Libralato, S., Capezzuto, F., (2019). Ecosystem functioning of two marine food webs in the North-Western Ionian Sea (Central Mediterranean Sea). *Ecol. Evol.*, 9, 10198–10212. doi: 10.1002/ece3.552

Ricci P., Manea E., Cipriano G., *et al.* (2021). Addressing cetacean–fishery interactions to inform a deep-sea ecosystem-based management in the Gulf of Taranto (northern Ionian Sea, central Mediterranean Sea). *J. Mar. Sci. Eng.*, 9(8), 872. https://doi.org/ 10.3390/jmse9080872.

Sartor, P., Carbonara, P., Lucchetti, A., Sabatella, E.C. (2016). Indagine conoscitiva sullo scarto della pesca alle specie demersali nei mari italiani. Valutazioni propedeutiche per l’implementazione delle disposizioni comunitarie in tema di obbligo di sbarco. *Quaderni Nisea*, n. 1: 40 pp - ISBN 978-88-94-1553-1-0

Sepulveda, C. A., Heberer, C., Aalbers, S. A., Spear, N., Kinney, M., Bernal, D., Kohin, S. (2015). Post-release survivorship studies on common thresher sharks (*Alopias vulpinus*) captured in the southern California recreational fishery. *Fish. Res.*, 161, 102–108. https://doi.org/10.1016/j.fishres.2014.06.014

Sims, D.W. (2008). Chapter 3 sieving a living: a review of the biology, ecology and conservation status of the plankton-feeding basking shark C*etorhinus maximus. Adv. Mar. Biol.*, 54: 171-220. https://doi.org/10.1016/S0065-2881(08)00003-5

Sims, D.W., and Merrett, D.A. (1997). Determination of zooplankton characteristics in the presence of surface feeding basking sharks *Cetorhinus maximus*. *Mar. Ecol. Progr. Ser.*, 158, 297–302

Sperone, E., Parise, G., Leone, A., et al. (2012). Spatiotemporal patterns of distribution of large predatory sharks in Calabria (central Mediterranean, southern Italy). *Acta Adriatica*, 53, 13-24

Teo, S.L.H., Rodriguez, E.G., Sosa-Nishizaki, O. (2018). Status of common thresher sharks, *Alopias vulpinus*, along the west coast of North America: update stock assessment based on alternative life history. Southwest Fisheries Science Center (U.S.). NOAA technical memorandum NMFS; NOAA-TM-NMFS-SWFSC; 595; https://doi.org/10.7289/v5/tm-swfsc-595

Tsagarakis, K., Palialexis, A., Vassilopoulou V. (2014). Mediterranean fishery discards: review of the existing knowledge. *ICES J. Mar. Sci.*, 71(5), 1219-1234., https://doi.org/10.1093/icesjms/fst074
